# Orthopaedic Trauma in Patients with Documented Alcohol Use: Injury Mechanisms, Clinical Characteristics, and Geriatric Vulnerability

**DOI:** 10.3390/medsci14040504

**Published:** 2026-08-21

**Authors:** Irina Sirbu, Bianca-Ana Dmour, Stefan-Dragos Tîrnovanu, Bogdan Puha, Eliza-Geanina Cogian, Mariana Zubenschi, Alexandru Filip, Ioana-Dana Alexa, Mihaela-Camelia Tîrnovanu, Popescu Dragos-Cristian, Adrian-Claudiu Carp, Awad Dmour

**Affiliations:** 1 Grigore T. Popa University of Medicine and Pharmacy, 700115 Iasi, Romania; sirbu_irina@d.umfiasi.ro (I.S.); bianca-ana.dmour@umfiasi.ro (B.-A.D.); stefan-dragos.tirnovanu@umfiasi.ro (S.-D.T.); alexandru-filip@umfiasi.ro (A.F.); ioana.alexa@umfiasi.ro (I.-D.A.); mihaela.tirnovanu@umfiasi.ro (M.-C.T.); dragos.popescu@umfiasi.ro (P.D.-C.); adrian-claudiu.carp@umfiasi.ro (A.-C.C.); dmour-awad@umfiasi.ro (A.D.); 2 Orthopaedic and Traumatology Clinic, ‘Sf. Spiridon’ Clinical Emergency Hospital, 700111 Iasi, Romania; 3 Cardiology Clinic, “St. Spiridon” County Clinical Emergency Hospital, 700111 Iasi, Romania; 4 “Ion Creangă” State Pedagogical University of Chișinău, 1 Ion Creangă Street, MD-2069 Chișinău, Moldova; cogian.eliza-geanina@upsc.md (E.-G.C.); zubenschi.mariana@upsc.md (M.Z.)

**Keywords:** alcohol use, alcohol withdrawal, orthopaedic trauma, geriatric trauma, proximal femoral fracture, low-energy falls, intensive care, hospital outcomes

## Abstract

Background: Alcohol-related conditions may influence both injury patterns and in-hospital management in orthopaedic trauma. This study evaluated the clinical characteristics, injury mechanisms, treatment patterns, and hospital outcomes of adults with acute orthopaedic trauma and documented alcohol use, with particular attention to geriatric vulnerability and alcohol withdrawal. Methods: This retrospective single-centre cohort included adults admitted between January 2018 and December 2025 with acute musculoskeletal trauma and an alcohol-related diagnosis during the same hospitalisation. Patients were classified according to their predominant recorded alcohol-related presentation. Geriatric patients, defined as those aged 65 years or older, were compared with younger adults. Injury mechanisms, comorbidities, operative treatment, intensive care unit involvement, hospital length of stay, mortality, and recorded hospitalisation costs were analysed. Results: The final cohort comprised 294 patients, including 87 geriatric patients. Geriatric patients more frequently sustained same-level or low-energy falls than younger adults (46.0% versus 25.6%; Holm-adjusted *p* = 0.005) and had a higher prevalence of proximal femoral fractures (40.2% versus 21.7%; OR 2.42, 95% CI 1.41 to 4.16). Any recorded ICU involvement was more frequent among geriatric patients, although prolonged ICU stays of 24 h or longer did not differ significantly between age groups. Alcohol withdrawal was documented in 46 patients and was associated with longer hospitalisation and a longer admission-to-surgery interval. In-hospital mortality occurred in 5 of 46 patients with documented withdrawal (10.9%) and 5 of 248 without withdrawal (2.0%; unadjusted OR 5.93, 95% CI 1.64 to 21.37; *p* = 0.010). Conclusions: Orthopaedic trauma patients with documented alcohol use represent a clinically heterogeneous population. Geriatric patients showed greater vulnerability to low-energy trauma, proximal femoral fracture, comorbidity, and intensive care involvement, while alcohol withdrawal identified patients with a more complex hospital course. Early recognition of withdrawal risk and enhanced inpatient safety measures may improve orthopaedic care. These findings support careful assessment of alcohol-related risk, early recognition of withdrawal, and heightened inpatient safety precautions.

## 1. Introduction

Alcohol consumption represents an important and potentially modifiable factor in traumatic injury. Acute intoxication may impair judgement, coordination, balance, reaction time, and risk perception, thereby increasing susceptibility to falls, interpersonal violence, and road-traffic injuries. Chronic harmful alcohol use may additionally coexist with malnutrition, hepatic dysfunction, neurological impairment, reduced bone quality, and diminished physiological reserve. These factors may influence not only the mechanism and severity of injury, but also the clinical course after hospital admission. In trauma populations, alcohol-related diagnoses and alcohol withdrawal syndrome have been associated with greater resource utilisation, prolonged hospitalisation, infectious complications, and increased in-hospital morbidity [1,2].

The orthopaedic setting presents several specific challenges. Patients may require urgent fracture stabilisation while simultaneously experiencing acute intoxication, withdrawal, behavioural disturbance, or medical complications related to chronic alcohol exposure. Symptoms such as agitation, tachycardia, tremor, confusion, or impaired cooperation may complicate clinical assessment and may be difficult to distinguish from pain, traumatic brain injury, postoperative delirium, infection, or medication-related effects. Furthermore, altered cognition and impulsive mobilisation may compromise weight-bearing restrictions, external fixation devices, surgical wounds, or recently performed osteosynthesis. Recognition of alcohol use and withdrawal risk is therefore relevant to both medical management and the mechanical protection of the orthopaedic reconstruction [3,4]. Psychological morbidity may represent an additional dimension of vulnerability, as depression and post-traumatic stress symptoms are common after acute orthopaedic injury and may coexist with alcohol misuse, potentially complicating functional recovery and engagement with rehabilitation [5,6].

Older adults may represent a particularly vulnerable subgroup. Geriatric trauma is frequently associated with same-level falls and may reflect the interaction of impaired balance, sarcopenia, frailty, osteoporosis, cognitive impairment, polypharmacy, and multiple chronic diseases. Alcohol exposure may further affect postural stability and judgement, while chronic harmful use may contribute to nutritional and skeletal deterioration. Consequently, an apparently low-energy mechanism can result in a major fracture, loss of mobility, prolonged rehabilitation, or institutional dependence. Nevertheless, the demographic profile, injury mechanisms, treatment patterns, and hospital outcomes of geriatric patients with documented alcohol use have not been adequately characterised in many orthopaedic trauma settings [7,8].

Road-traffic injuries constitute another clinically relevant presentation. Alcohol involvement may be documented among drivers, passengers, cyclists, pedestrians, or other road users, but its presence at admission should not automatically be interpreted as proof that alcohol caused the collision. Road-traffic injuries may nevertheless differ from other mechanisms because they frequently involve higher-energy trauma, multiple anatomical regions, open fractures, and greater critical care requirements [9]. Characterising the road-user category, intoxication status, injury pattern, treatment, and hospital outcome may therefore provide useful complementary information within a broader orthopaedic trauma cohort.

The present study aimed to characterise the injury mechanisms, orthopaedic diagnoses, clinical features, treatment patterns, and in-hospital outcomes of adults admitted with acute orthopaedic trauma and documented alcohol use between 2018 and 2025. The principal analysis compared geriatric patients, defined as those aged 65 years or older, with younger adults. Secondary objectives were to describe the distribution of alcohol-related clinical presentations, including acute intoxication, harmful use, dependence, and withdrawal; to characterise road-traffic injuries and the proportion accompanied by documented acute intoxication; and to evaluate operative treatment, hospital length of stay, intensive care involvement, and in-hospital mortality.

## 2. Materials and Methods

### 2.1. Study Design and Setting

This retrospective, single-centre observational cohort study was conducted in the Clinic of Orthopaedics and Traumatology at Sf. Spiridon County Clinical Emergency Hospital, Iași, Romania. Adult patients admitted between 1 January 2018 and 31 December 2025 were considered for inclusion.

Data were retrieved from the hospital electronic information system and included demographic characteristics, admission and discharge information, diagnostic codes, clinical summaries, operative procedures, laboratory findings, intensive care involvement, and discharge status. The study was reported in accordance with the STROBE statement and the RECORD extension for studies based on routinely collected health data [10,11].

### 2.2. Study Population

Patients were identified through an electronic search of hospital records using the prespecified alcohol-related ICD-10 codes among admissions occurring between 1 January 2018 and 31 December 2025. In routine clinical practice, these codes were assigned when an alcohol-related problem was clinically documented or when available laboratory findings, including blood ethanol testing, supported an alcohol-related diagnosis. Blood ethanol testing was not used as an independent case-finding mechanism in the absence of a qualifying alcohol-related diagnostic code. Systematic alcohol screening was not performed; consequently, patients whose alcohol use was not recognized, documented, or coded were not captured by the study.

Patients were eligible if they were aged 18 years or older, had been admitted with an acute traumatic musculoskeletal injury, and had documented alcohol use or an alcohol-related disorder during the same hospitalisation.

Acute orthopaedic trauma included fractures, dislocations, crushing injuries, traumatic amputations, and other acute musculoskeletal injuries involving the spine, pelvis, or extremities. Associated injuries involving other anatomical regions were recorded when present.

Admissions exclusively related to elective orthopaedic treatment, degenerative musculoskeletal disease, non-traumatic avascular necrosis, chronic implant-related complications without a new injury, or other non-traumatic conditions were excluded.

The individual patient was considered the unit of analysis. When more than one eligible admission was identified for the same patient, the first eligible admission was retained for the main analysis.

### 2.3. Identification of Documented Alcohol

Alcohol-related status was established from the diagnostic codes and the corresponding clinical information available in the electronic patient records. Eligible ICD-10 codes included Z72.1, F10.0–F10.9, T51.0, T51.9, and Y90.0–Y90.9. For comparative analyses, patients were assigned to one mutually exclusive category representing the predominant recorded alcohol-related presentation during the index admission: alcohol withdrawal, acute intoxication or toxic alcohol effect, harmful use or alcohol dependence, or other or unspecified documented alcohol use.

When more than one alcohol-related condition was recorded, a prespecified hierarchy was applied. Withdrawal took precedence over acute intoxication, followed by harmful use or dependence and other or unspecified documented alcohol use. This hierarchy was used solely to prevent multiple classification of the same patient and was not intended to represent a scale of alcohol consumption or disorder severity.

Alcohol withdrawal was considered present when explicitly recorded in the diagnostic coding or clinical documentation; a formal CIWA-Ar threshold was not required. Acute intoxication was based on an acute alcohol-related or toxic-effect diagnosis or explicit clinical documentation, with blood ethanol testing used as supporting evidence when available. Harmful alcohol use or dependence was identified from the corresponding diagnostic coding and clinical documentation. These categories therefore represent recorded clinical presentations rather than systematic measurements of alcohol consumption or disorder severity.

Raw alcohol-related diagnostic codes were also examined non-mutually exclusively before application of the analytical hierarchy in order to describe diagnostic overlap.

### 2.4. Study Variables

Demographic variables included age, sex, and rural or urban residence. Patients aged 65 years or older were classified as geriatric.

Clinical variables included admission type, injury mechanism, principal orthopaedic diagnosis, anatomical injury location, open fracture, polytrauma, operative treatment, hospital length of stay, intensive care involvement, discharge status, and in-hospital mortality. The recorded hospitalisation cost for each episode of care was extracted from the hospital electronic database and analysed in nominal Romanian lei. Annual unfiltered section activity was obtained from institutional administrative reports and included all patient episodes managed by the Orthopaedics and Traumatology Emergency Section. These data were used solely to contextualise the annual size of the analytical cohort.

Polytrauma was considered present when explicitly documented in the diagnostic coding or clinical record. Injury Severity Score and the Berlin polytrauma criteria were not systematically available; accordingly, this variable is referred to as documented polytrauma throughout the manuscript.

ICU involvement was defined as any electronically recorded episode under the intensive care service. Because the electronic system also records brief perioperative and postoperative surveillance, ICU involvement was further characterised according to duration. Episodes lasting less than 24 h were classified as brief ICU surveillance, whereas episodes lasting 24 h or longer were classified as prolonged ICU stays. When both timestamps were available, the temporal relationship between ICU involvement and the recorded surgical intervention was also evaluated. Planned versus unplanned ICU care and the specific indication for ICU involvement could not be reliably distinguished from the routinely collected data.

Recorded comorbidities included cardiovascular disease, chronic liver disease, and neurological or cognitive disease. A descriptive composite variable indicated the presence of at least one of these selected comorbidity categories. Selected individual diagnoses, including hypertension (I10–I15), atrial fibrillation or flutter (I48), heart failure (I50), anaemia (D50–D64), and osteoporosis (M80–M82), were evaluated in exploratory diagnosis-level analyses.

Major comorbidities were identified from the admission, secondary, and discharge diagnoses. These included cardiovascular, renal, hepatic, respiratory, neurological, metabolic, infectious, and psychiatric conditions. Among operatively treated patients, time to surgery was calculated in hours from the recorded hospital admission timestamp to the recorded intervention timestamp. The exact onset of alcohol withdrawal relative to surgery was not consistently timestamped in the routine clinical records.

### 2.5. Injury Mechanisms

The mechanism of injury was determined from diagnostic codes and the available clinical documentation. Injuries were classified as same-level or low-energy falls, falls from height, road-traffic injuries, bicycle-related injuries, interpersonal violence, other traumatic mechanisms, or unspecified mechanisms.

Road-traffic injuries required explicit documentation of a collision or transport-related event. Road-user status was classified as driver, passenger, pedestrian, cyclist, other road user, or unspecified.

Among patients with road-traffic injuries, acute intoxication was considered present only when supported by a specific diagnosis, blood alcohol documentation, or explicit clinical description. Documented intoxication was not interpreted as proof that alcohol caused the collision.

### 2.6. Outcomes

The principal analysis examined the distribution of injury mechanisms and orthopaedic injury characteristics in the overall cohort and compared geriatric patients with younger adults.

Secondary outcomes included operative treatment, hospital length of stay, any recorded ICU involvement, prolonged ICU stay of 24 h or longer, documented alcohol withdrawal, open fracture, documented polytrauma, discharge status, and in-hospital mortality. Road-traffic injuries were analysed separately according to road-user category, alcohol-related presentation, injury characteristics, treatment, and hospital outcome.

The age-group comparison of injury mechanisms and orthopaedic injury characteristics constituted the principal analysis. Diagnosis-level comparisons, withdrawal-related analyses, road-traffic subgroup analyses, multivariable models, and hospitalisation-cost analyses were considered exploratory secondary analyses.

### 2.7. Statistical Analysis

Statistical analyses were performed using IBM SPSS Statistics, version 27.0 (IBM Corp., Armonk, NY, USA). Categorical variables were summarised as frequencies and percentages, while continuous variables were reported as means with standard deviations or medians with interquartile ranges, according to their distribution.

Comparisons between geriatric patients and younger adults were performed using Pearson’s chi-square test or Fisher’s exact test for categorical variables and the independent-samples *t*-test or Mann–Whitney U test for continuous variables, as appropriate. Overall differences in the distributions of injury mechanisms and predominant alcohol-related presentations were assessed using Pearson’s chi-square test, with Cramér’s V reported as the corresponding effect-size measure. When a significant overall difference was identified, category-specific post hoc comparisons were performed using two-sided Fisher’s exact tests, with *p* values adjusted according to the Holm method.

Selected diagnosis-level comparisons were considered exploratory and were performed using two-sided Fisher’s exact tests, with *p* values adjusted using the Holm method.

Odds ratios with 95% confidence intervals were calculated for selected binary outcomes. Key proportions were reported with 95% confidence intervals. Road-traffic and transport-related injuries were analysed descriptively because of the limited subgroup size. Annual hospitalisation costs were reported as cumulative nominal values and as medians with interquartile ranges.

Exploratory multivariable logistic regression analyses were performed for ICU involvement and proximal femoral fracture. The ICU model included age group, sex, open fracture, polytrauma, proximal femoral fracture, alcohol withdrawal, and the presence of at least one selected comorbidity. The proximal femoral fracture model included age group, sex, same-level or low-energy fall, harmful alcohol use or dependence, and the presence of at least one selected comorbidity. Covariates were selected before model fitting according to clinical relevance and the available number of outcome events, without automated variable-selection procedures. Multicollinearity was assessed using variance inflation factors. Model discrimination was evaluated using the area under the receiver operating characteristic curve, and calibration was explored using the Hosmer–Lemeshow goodness-of-fit test. As a sensitivity analysis, age was entered as a continuous variable, with effect estimates expressed per 10-year increase, replacing the dichotomous geriatric age variable while the remaining covariates were unchanged.

Complete data were available for the variables included in the principal age-group comparisons and multivariable models. The unspecified injury-mechanism category reflected the information recorded in the source documentation and was not treated as missing data. No a priori sample-size calculation was performed because all eligible patients identified during the prespecified eight-year study period were included.

### 2.8. Selection of Illustrative Clinical Case

One case from the analytical cohort was selected to illustrate the orthopaedic consequences of withdrawal-related behavioural disturbance, secondary in-hospital trauma, and staged fracture management. The case was included solely for clinical illustration and was not analysed separately from the cohort.

### 2.9. Ethical Approval

The study was conducted in accordance with the Declaration of Helsinki and was approved by the Ethics Committee of Sf. Spiridon County Clinical Emergency Hospital, Iași, Romania (Approval No. 54, 6 April 2026).

Written informed consent was obtained from the patients whose individual clinical cases or medical images are presented in the manuscript.

## 3. Results

### 3.1. Cohort Selection and Overall Characteristics

**Figure 1:**
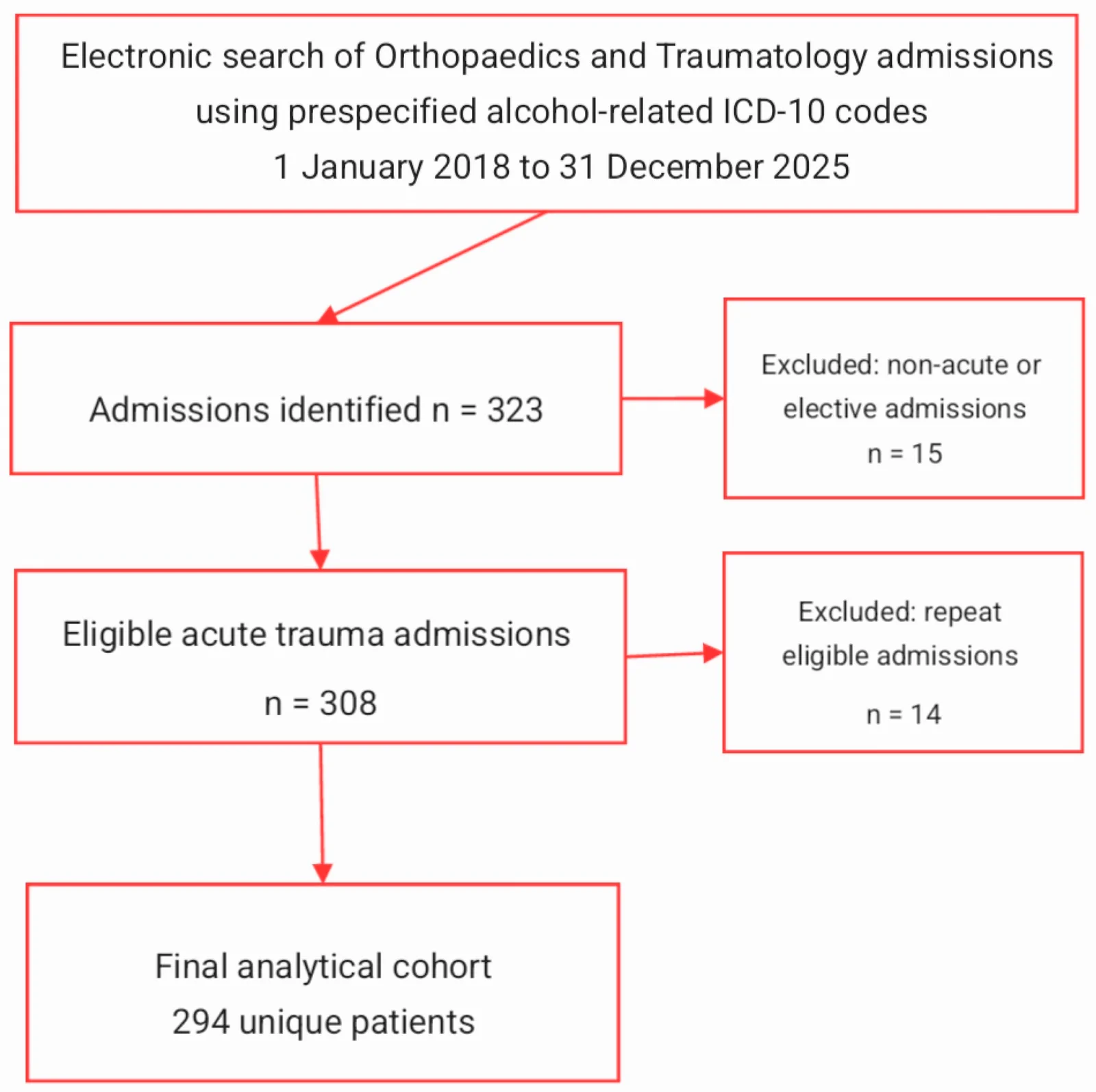
Cohort selection process. Patients were identified through an electronic search of Orthopaedics and Traumatology admissions using prespecified alcohol-related ICD-10 codes. After exclusion of 15 non-acute or elective admissions and 14 repeat eligible admissions, 294 unique patients were included in the final analysis.

A total of 323 admissions were screened. After excluding 15 non-acute or elective admissions and 14 repeat admissions, 294 unique patients were included in the final analysis (Figure 1). The mean age was 56.3 ± 13.4 years, and 87 patients (29.6%) were aged 65 years or older. The overall demographic, alcohol-related, injury, and hospital characteristics are presented in Table 1 and Table 2.

During the study period, 13 patients (4.4%) had at least one additional eligible alcohol-related trauma admission at the same institution. Fourteen repeat admissions were excluded from the main patient-level analysis, with a median interval of 271 days (IQR 24 to 722) from the first eligible admission. The primary outcome analysis was restricted to the index hospitalisation. Recurrent eligible trauma admissions could be identified only when patients returned to the same institution, while all-cause readmissions, admissions to other institutions, and systematic post-discharge outcomes were unavailable.

### 3.2. Demographic and Alcohol-Related Characteristics According to Age Group

Geriatric patients included a lower proportion of men and had a higher prevalence of recorded cardiovascular comorbidity than younger adults. At least one cardiovascular, chronic liver, or neurological or cognitive comorbidity was documented in 51 of 87 geriatric patients (58.6%) and 66 of 207 younger adults (31.9%; *p* < 0.001).

In exploratory diagnosis-level analyses, hypertension, atrial fibrillation or flutter, anaemia, and osteoporosis were more frequently recorded among geriatric patients. Hypertension was present in 25 of 87 geriatric patients (28.7%) and 24 of 207 younger adults (11.6%; Holm-adjusted *p* = 0.003), while atrial fibrillation or flutter was recorded in 10 geriatric patients (11.5%) and four younger adults (1.9%; Holm-adjusted *p* = 0.005). Anaemia was documented in 23 geriatric patients (26.4%) and 24 younger adults (11.6%; Holm-adjusted *p* = 0.008), and osteoporosis in 10 geriatric patients (11.5%) and five younger adults (2.4%; Holm-adjusted *p* = 0.008). Heart failure was recorded in four geriatric patients (4.6%) and three younger adults (1.4%), without a significant difference after Holm adjustment (*p* = 0.201).

Blood ethanol testing was documented in 157 patients (53.4%), including 70 of 80 patients (87.5%) classified with acute alcohol intoxication. Before application of the mutually exclusive hierarchy, Z72.1 was recorded in 267 patients (90.8%), at least one F10 diagnosis in 53 (18.0%), a T51 diagnosis in five (1.7%), and a Y90 code in four (1.4%); 41 patients (13.9%) had more than one alcohol-related ICD-10 code.

Within the F10 group, F10.0 was recorded in 3 patients, F10.1 in 1, F10.2 in 2, F10.3 in 13, F10.4 in 5, F10.5 in 2, and F10.9 in 35; these frequencies were non-mutually exclusive.

**Figure 2:**
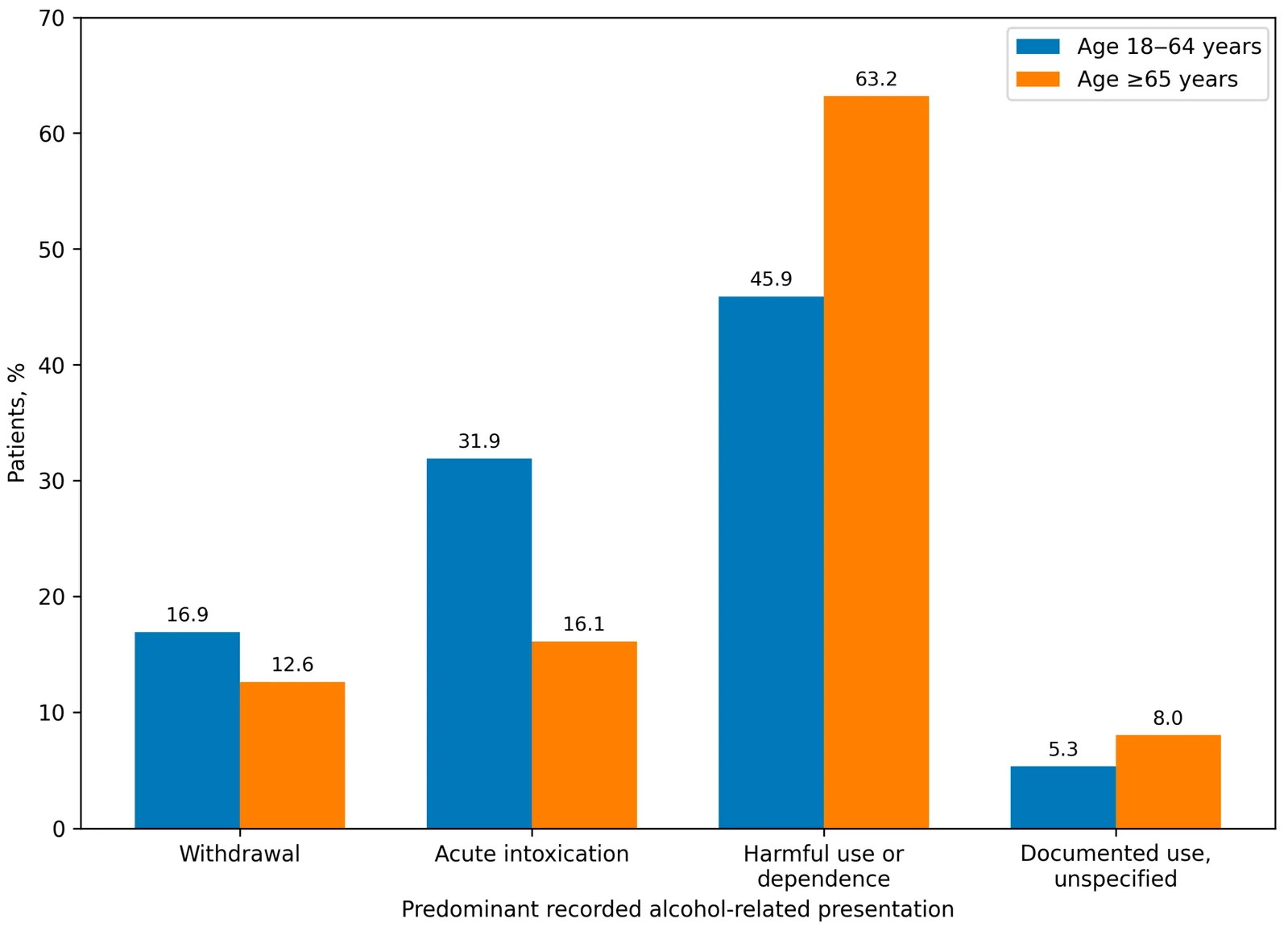
Distribution of predominant recorded alcohol-related presentations according to age group. Acute alcohol intoxication was less frequent, whereas harmful alcohol use or dependence was more frequent, among geriatric patients.

The distribution of predominant recorded alcohol-related presentations differed between age groups (χ^2^(3) = 10.68, *p* = 0.014; Cramér’s V = 0.191). Acute alcohol intoxication was less frequently recorded among geriatric patients, whereas harmful alcohol use or dependence was more frequent. No significant age-related differences were observed for alcohol withdrawal or documented alcohol use without a specific clinical phenotype (Figure 2).

### 3.3. Injury Mechanisms and In-Hospital Outcomes According to Age Group

The distribution of injury mechanisms differed significantly between age groups (*p* = 0.001; Cramér’s V = 0.245), as shown in Table 2. Same-level or low-energy falls were more frequent among geriatric patients, while interpersonal violence and other traumatic mechanisms were less common.

The 80 proximal femoral fractures comprised 19 femoral neck fractures, 55 trochanteric fractures, and six subtrochanteric fractures. Among younger and geriatric patients, respectively, there were 11 versus eight femoral neck fractures, 31 versus 24 trochanteric fractures, and three versus three subtrochanteric fractures. A recorded operative procedure was present in 16 of 19 femoral neck fractures, 51 of 55 trochanteric fractures, and five of six subtrochanteric fractures. Proximal femoral fractures were identified in 35 geriatric patients (40.2%), compared with 45 younger adults (21.7%). Geriatric patients consequently had more than twice the odds of sustaining a proximal femoral fracture (OR 2.42, 95% CI 1.41 to 4.16; *p* = 0.002). Among 72 operatively treated patients with proximal femoral fractures, the median admission-to-surgery interval was 80.0 h (IQR 54.3 to 135.6). Three patients (4.2%) underwent surgery within 24 h and 16 (22.2%) within 48 h. The admission-to-surgery interval did not differ significantly between geriatric and younger patients (median 70.2 versus 88.3 h; *p* = 0.301).

Among the 20 geriatric patients without a recorded operative procedure, the principal injuries were pelvic or acetabular fractures in eight patients, proximal femoral fractures in five, humeral fractures in three, ankle fractures in two, and forearm and distal femoral fractures in one patient each. Reasons for non-operative management were not available as a uniform structured variable and were therefore not analysed comparatively.

Any recorded ICU involvement occurred in 117 patients (39.8%); however, 98 of these 117 episodes (83.8%) lasted less than 24 h and predominantly represented brief perioperative surveillance. The median duration of these brief episodes was 1.67 h (IQR 1.00 to 2.73). Among 96 patients with both a brief ICU episode and a recorded surgical timestamp, 80 (83.3%) entered ICU after surgery. Prolonged ICU stays of 24 h or longer occurred in 19 patients (6.5%), with no significant difference between geriatric and younger patients (4.6% versus 7.2%; *p* = 0.604). The median duration of prolonged ICU stays was 93.8 h (IQR 58.7 to 336.7). Among 17 patients with a prolonged ICU stay and an available surgical timestamp, the first ICU episode occurred after surgery in nine and before surgery in eight patients.

In exploratory multivariable analyses, geriatric age and proximal femoral fracture remained associated with any recorded ICU involvement after multivariable adjustment (Table 3). Geriatric patients had higher adjusted odds of ICU involvement than younger adults (adjusted OR 1.80, 95% CI 1.03 to 3.13; *p* = 0.038), while proximal femoral fracture was associated with almost threefold higher odds of ICU involvement (adjusted OR 2.96, 95% CI 1.66 to 5.27; *p* < 0.001). the presence of at least one selected comorbidity remained associated with proximal femoral fracture after multivariable adjustment.

In the proximal femoral fracture model, geriatric age (adjusted OR 1.84, 95% CI 1.02 to 3.31; *p* = 0.042), same-level or low-energy fall (adjusted OR 2.12, 95% CI 1.21 to 3.71; *p* = 0.009), and the presence of at least one selected comorbidity (adjusted OR 1.96, 95% CI 1.13 to 3.42; *p* = 0.018) remained associated after multivariable adjustment with the outcome. The findings remained consistent when age was modelled continuously. Overall, operative treatment was recorded in 42 of 45 younger patients (93.3%) and 30 of 35 geriatric patients (85.7%) with proximal femoral fractures.

No evidence of problematic multicollinearity was identified, with variance inflation factors below 2.1 in both models. Discrimination was modest for both the ICU model (AUC 0.686) and the proximal femoral fracture model (AUC 0.697). The Hosmer–Lemeshow test indicated no evidence of poor calibration for the ICU model (*p* = 0.362), whereas calibration was limited for the proximal femoral fracture model (*p* = 0.005); the latter should therefore be interpreted as an exploratory descriptive model rather than a causal risk model.

### 3.4. Road-Traffic and Transport-Related Injuries

Of the 28 patients included in the broader road, transport, or bicycle-related category, 23 met the prespecified definition of a road-traffic or transport event and formed the dedicated subgroup. The remaining five patients had bicycle-related injuries without explicit documentation of a road-traffic event. The subgroup was predominantly male, and pedestrians represented the most frequent road-user category (Table 4). Acute alcohol intoxication was documented in 60.9% of cases (95% CI 40.8% to 77.8%). Polytrauma and open fractures were frequent, and two in-hospital deaths were recorded. Given the limited subgroup size, these findings were analysed descriptively.

### 3.5. Documented Alcohol Withdrawal and Perioperative Course

Alcohol withdrawal was documented in 46 patients (15.6%). Patients with withdrawal had a longer median hospital stay than those without withdrawal, 9.5 days (IQR 7.0 to 12.4) versus 7.5 days (IQR 4.7 to 11.2), respectively (*p* = 0.015).

In-hospital mortality occurred in 5 of 46 patients with withdrawal (10.9%) and in 5 of 248 patients without withdrawal (2.0%) (OR 5.93, 95% CI 1.64 to 21.37; *p* = 0.010). Because only 10 deaths occurred in the entire cohort, this unadjusted association should be interpreted cautiously.

Among the 249 operatively treated patients, the median admission-to-surgery interval was 59.6 h (IQR 27.3 to 120.5). Patients with documented alcohol withdrawal during the index hospitalisation had a longer interval than those without documented withdrawal, with median values of 95.8 h (IQR 55.9 to 142.5) and 55.5 h (IQR 23.3 to 106.3), respectively (*p* = 0.002). Because the timing of withdrawal onset relative to surgery was not systematically recorded, this association does not establish that withdrawal caused surgical delay.

Prolonged ICU stays of 24 h or longer occurred in 7 of 46 patients with documented withdrawal (15.2%) and 12 of 248 patients without withdrawal (4.8%; unadjusted OR 3.53, 95% CI 1.31 to 9.52; *p* = 0.017). Given the limited number of prolonged ICU events, no multivariable analysis was performed for this outcome.

### 3.6. Annual Cohort Distribution and Nominal Hospitalisation Costs

**Figure 3:**
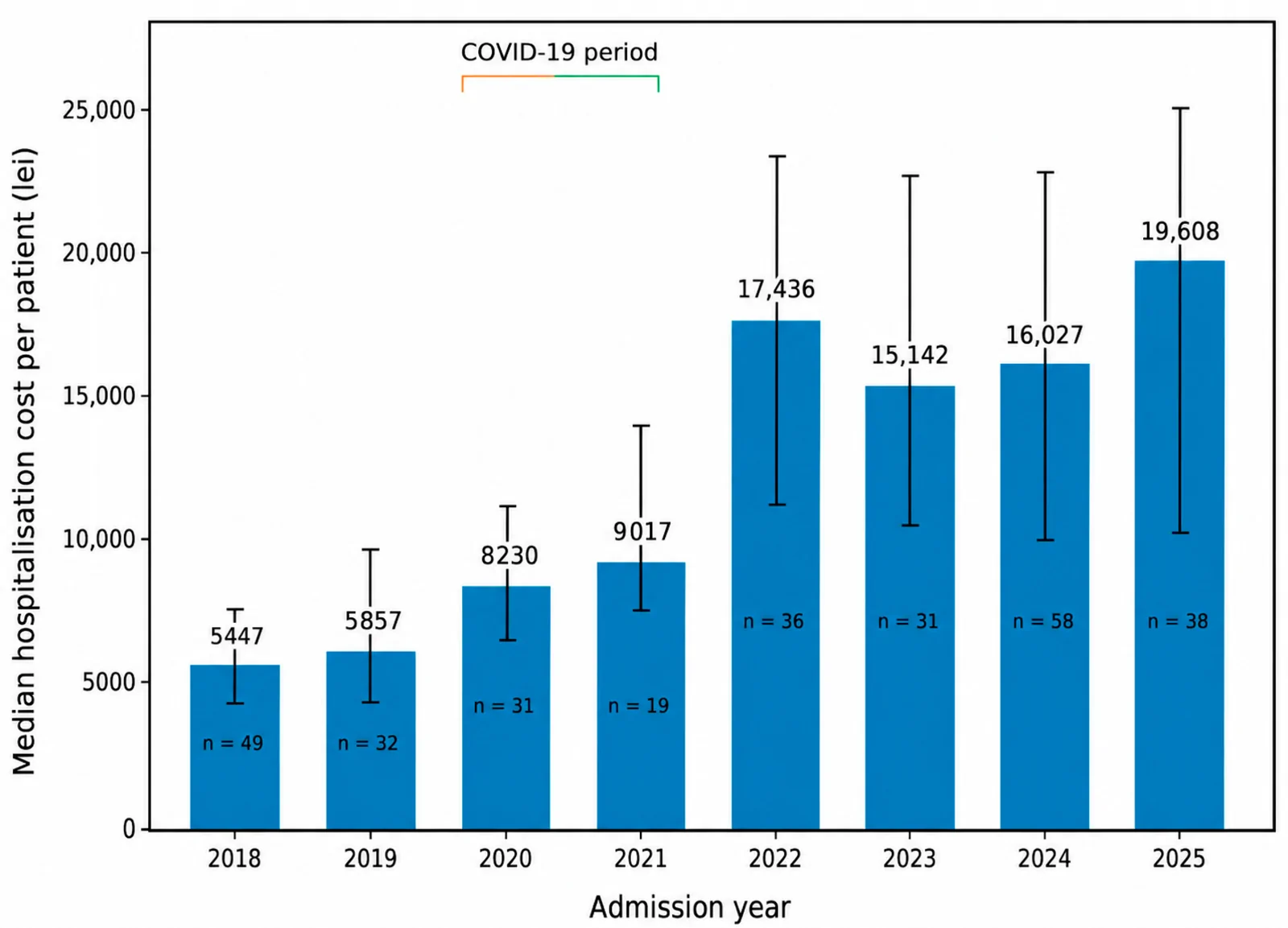
Annual cohort size and median nominal recorded hospitalisation cost per patient between 2018 and 2025. The COVID-19 period is indicated for temporal context. Costs represent nominal values recorded in the hospital database and were not adjusted for inflation, reimbursement changes, implant or consumable prices, or changes in hospital accounting practices.

Annual cohort size and median recorded hospitalisation costs are presented in Figure 3. The lower numbers of included patients in 2020 and 2021 coincided with a broader reduction in activity within the Orthopaedics and Traumatology Emergency Section. The total number of hospitalised patient episodes decreased from 4101 in 2018 and 4057 in 2019 to 2069 in 2020 and 2375 in 2021, before increasing to 2939 in 2022, 3364 in 2023, 3403 in 2024, and 3361 in 2025.

The cumulative nominal hospitalisation cost for the cohort was 5,456,655.28 lei, with a median of 10,614.31 lei per patient (IQR 6362.10 to 19,546.35). The annual median nominal cost ranged from 5446.91 lei in 2018 to 19,607.71 lei in 2025. Temporal cost differences were analysed descriptively because the recorded values were not adjusted for inflation, changes in reimbursement, implant and consumable prices, or hospital accounting practices.

### 3.7. Representative Clinical Case

**Figure 4:**
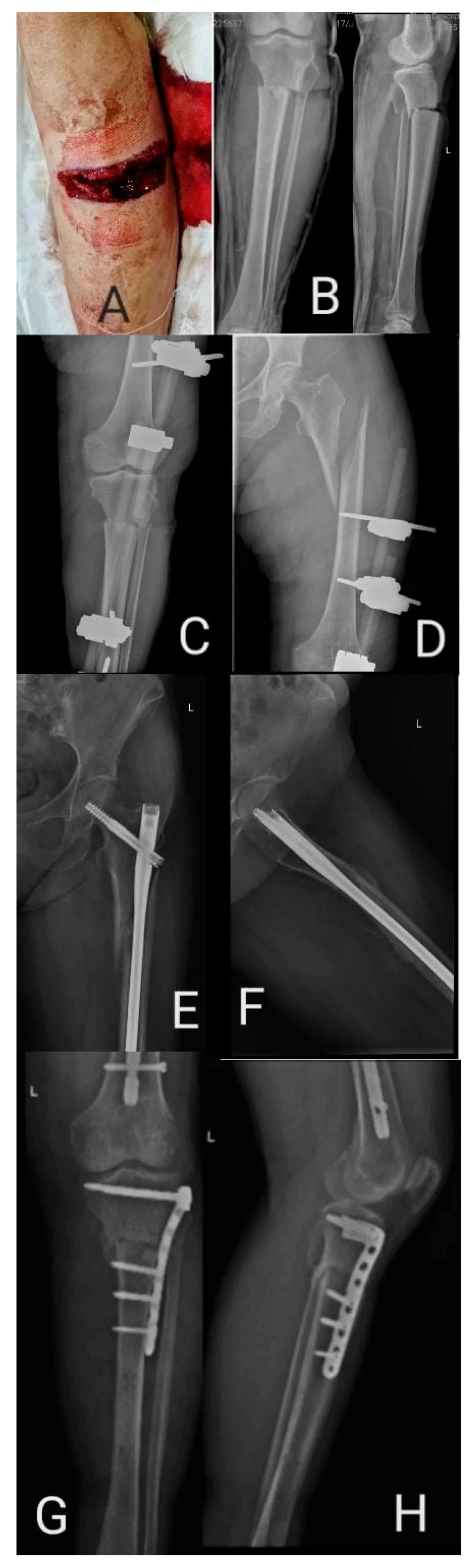
Staged management of an open proximal tibial fracture complicated by alcohol withdrawal-related in-hospital trauma. (**A**) Clinical appearance of the contaminated open wound associated with the Gustilo–Anderson type IIIA proximal tibial fracture. (**B**) Initial anteroposterior and lateral radiographs of the proximal tibial fracture. (**C**) Temporary stabilisation using a knee-spanning external fixator. (**D**) Subtrochanteric femoral fracture sustained during an in-hospital fall, with the external fixator remaining in situ. (**E**,**F**) Anteroposterior and lateral radiographs after closed reduction and fixation of the subtrochanteric fracture with a long Gamma nail. (**G**,**H**) Anteroposterior and lateral radiographs after definitive fixation of the proximal tibial fracture with an angular-stable plate.

A female patient was admitted after a lawnmower injury sustained while both she and her partner, who was operating the machine, were under the influence of alcohol. The injury resulted in a deeply contaminated open fracture of the proximal left tibia and fibula with extensive soft-tissue damage, classified as Gustilo–Anderson type IIIA. Initial management consisted of surgical debridement, irrigation, antibiotic therapy, and temporary external fixation. On the third hospital day, the patient developed alcohol withdrawal with agitation and confusion, left the bed without assistance, and sustained an in-hospital fall resulting in a left subtrochanteric femoral fracture. The femoral fracture was treated by closed reduction and fixation with a long Gamma nail, followed two weeks later by definitive fixation of the tibial fracture using an angular-stable plate. Postoperative evolution was favourable, with satisfactory radiographic alignment and implant positioning (Figure 4).

## 4. Discussion

The present findings identify two clinically distinct patterns of vulnerability among orthopaedic trauma patients with documented alcohol use. First, geriatric patients showed a predominance of same-level or low-energy falls and proximal femoral fractures, together with a greater burden of recorded comorbidity and more frequent recorded ICU involvement, largely reflecting brief perioperative surveillance rather than prolonged ICU stays. This pattern is consistent with the broader geriatric trauma literature, in which impaired balance, multimorbidity, skeletal fragility, and reduced physiological reserve increase the consequences of apparently minor mechanisms of injury [12,13]. Second, alcohol withdrawal was associated with longer hospitalisation and higher unadjusted in-hospital mortality. Previous trauma and operative cohorts have similarly associated alcohol withdrawal with increased complications, resource utilisation, prolonged hospitalisation, and mortality [1,2]. In the orthopaedic setting, withdrawal may have an additional mechanical relevance because agitation, confusion, and unsafe mobilisation can threaten wounds, external fixation constructs, and recently performed osteosynthesis [4,14]. Nevertheless, the mortality association observed in the present study should be regarded as exploratory because of the limited number of deaths and the retrospective identification of withdrawal from routinely recorded clinical diagnoses.

### 4.1. Geriatric Vulnerability and Injury Pattern

The predominance of same-level or low-energy falls and proximal femoral fractures among geriatric patients is consistent with the established pattern of trauma in older adults. Falls in this population are usually multifactorial and reflect the combined effects of impaired balance, reduced muscle strength, gait disturbance, cognitive impairment, polypharmacy, comorbidity, and skeletal fragility [12,15]. In the present cohort, the higher prevalence of recorded cardiovascular and other selected comorbidities among geriatric patients supports the interpretation that apparently minor trauma occurred within a background of reduced physiological reserve. Alcohol exposure may further increase vulnerability through impaired postural control and judgement, while prolonged harmful consumption may adversely affect nutrition, bone remodelling, and fracture susceptibility [8,14]. The absence of an age-related difference in prolonged ICU stays suggests that the higher frequency of recorded ICU involvement among geriatric patients largely reflected perioperative monitoring rather than a demonstrably greater requirement for sustained intensive care.

The consistency of the associations when age was analysed as a continuous variable suggests that the findings were not solely dependent on the conventional 65-year threshold. However, because bone mineral density, frailty, medication use, nutritional status, and the quantity or duration of alcohol consumption were not available, the observed association between geriatric age and proximal femoral fracture should not be attributed specifically to alcohol-related skeletal deterioration.

### 4.2. Clinical Significance of Alcohol Withdrawal in Orthopaedic Trauma

The association between documented alcohol withdrawal and both prolonged hospitalisation and increased in-hospital mortality represents one of the most clinically relevant findings of this study. Similar associations have been reported in large trauma cohorts, in which alcohol withdrawal syndrome was linked to longer hospital stay, greater intensive care requirements, more complications, and increased resource utilisation [1,2]. In operative trauma patients, withdrawal may reflect not only the physiological consequences of chronic alcohol dependence but also a more complex clinical course involving autonomic instability, delirium, seizures, infection, and difficulties with postoperative care [1,2,14]. Patients with documented withdrawal also more frequently experienced prolonged ICU stays, although this association was unadjusted and based on only 19 prolonged ICU events.

Because withdrawal onset relative to surgery was not consistently timestamped, the longer admission-to-surgery interval observed in patients with withdrawal cannot be interpreted as evidence that withdrawal itself delayed surgery.

These complications have particular relevance in orthopaedic practice. Agitation, confusion, tremor, and impaired cooperation may interfere with weight-bearing restrictions and increase the risk of unsupervised mobilisation, falls, wound disruption, or compromise of external fixation and recent osteosynthesis [4,14]. The representative case illustrates this mechanical dimension, as withdrawal-related agitation preceded an in-hospital fall and a second major fracture. Nevertheless, the present observational data cannot establish that withdrawal directly caused the excess mortality, and the estimate was based on only ten deaths without multivariable adjustment. The mortality finding should therefore be interpreted as an important safety signal rather than an independent prognostic effect.

### 4.3. Age-Related Patterns of Alcohol-Related Presentation

The lower frequency of recorded acute intoxication and the higher frequency of harmful alcohol use or dependence among geriatric patients may reflect differences in the clinical presentation and recognition of alcohol-related disorders across age groups. In older adults, chronic alcohol use may coexist with multimorbidity, cognitive impairment, medication effects, and functional decline, which can obscure or modify the typical presentation of intoxication or dependence [3,16]. Furthermore, routine diagnostic coding may capture chronic harmful use more readily when it is already established in the medical history, whereas acute intoxication depends more strongly on timely clinical recognition, explicit documentation, or blood ethanol testing. Structured instruments such as AUDIT or AUDIT-C can improve the identification of unhealthy alcohol use beyond routine clinical judgement, but such screening was not systematically available in the present cohort [16].

The overlap observed in the original diagnostic coding further emphasises that the mutually exclusive categories were analytical constructs used to prevent double counting. In particular, the harmful use or dependence category should be interpreted as the predominant recorded presentation in the absence of a higher-priority withdrawal or intoxication classification, rather than as a measure of greater or lesser alcohol-use severity. Therefore, these age-related differences should be interpreted as differences in the predominant recorded alcohol-related presentation, rather than as direct evidence of differences in alcohol quantity, frequency, or disorder severity.

### 4.4. Road-Traffic Injuries and Vulnerable Road Users

The road-traffic subgroup was characterised by a high frequency of documented acute intoxication, with pedestrians representing the largest road-user category. Acute alcohol exposure has a dose-dependent association with the risk of injury and traffic collision, partly through impaired judgement, reaction time, coordination, and risk perception [17]. The predominance of pedestrians is clinically relevant because these patients lack the physical protection afforded to vehicle occupants and may consequently sustain complex or multisystem injuries, as reflected by the frequency of polytrauma and open fractures in the present subgroup. Patterns of alcohol-related trauma may also vary according to local mobility, drinking behaviour, and broader social conditions, as illustrated by changes reported during COVID-19 restrictions in major trauma populations [18]. Nevertheless, the limited subgroup size, absence of a comparison group without documented alcohol use, and incomplete systematic blood ethanol testing prevent conclusions regarding attributable risk or causality. Documented intoxication should therefore be interpreted as a concurrent clinical finding rather than proof that alcohol caused the collision.

### 4.5. Clinical Implications for Inpatient Safety and Resource Use

The findings support a more systematic approach to identifying alcohol-related risk at the time of orthopaedic admission. Routine clinical documentation alone may fail to recognise patients at risk of withdrawal, particularly when pain, postoperative delirium, head injury, medication effects, or cognitive impairment produce overlapping symptoms. Brief screening instruments such as AUDIT-C can help identify unhealthy alcohol use, while the Prediction of Alcohol Withdrawal Severity Scale may assist in recognising patients at increased risk of complicated withdrawal [16,19]. CIWA-Ar may subsequentlv assist in symptom-based assessment of established withdrawal although its performance may be less reliable in acutely ill or injured hospitalised patients [20]. Patients considered at high risk may benefit from protocol-based monitoring, timely medical treatment, thiamine supplementation when clinically indicated, and early involvement of anaesthesia, intensive care, internal medicine, psychiatry, and nursing teams [4,14]. In orthopaedic wards, these measures should be accompanied by specific mechanical-safety precautions, including supervised mobilisation, reinforcement of weight-bearing restrictions, appropriate fall-prevention measures, and closer observation of patients whose agitation or confusion could compromise wounds, external fixation devices, or recent osteosynthesis [4,14].

Beyond the acute management of alcohol withdrawal, psychological distress may influence recovery after orthopaedic trauma [5,21,22]. Depression and post-traumatic stress symptoms are common after musculoskeletal injury, and their coexistence with alcohol misuse may further complicate pain, functional recovery, adherence to rehabilitation, and continuity of care [5,6,21]. Brief psychological screening and referral may therefore complement alcohol-withdrawal protocols when persistent distress is identified during hospitalisation or follow-up [23] Early supportive psychological approaches may be considered in distressed trauma patients, although evidence regarding the optimal implementation and effectiveness of Psychological First Aid remains limited [22].

Alcohol withdrawal may also contribute to increased resource utilisation through prolonged hospitalisation, critical care requirements, additional monitoring, and treatment of associated complications [1,2]. The lower number of included patients during 2020 and 2021 coincided with a marked reduction in overall section activity during the COVID-19 period. Changes in the frequency and composition of alcohol-related trauma were also reported by a major trauma centre during lockdown [18]. Similarly, the observed increase in recorded hospitalisation costs over time cannot be attributed solely to greater clinical complexity. The analysis used nominal values and did not account for inflation, reimbursement policies, implant and consumable prices, or changes in hospital accounting practices. Consequently, these data provide descriptive information on recorded inpatient expenditure rather than a formal health-economic assessment. Future studies incorporating inflation-adjusted costs, detailed resource categories, and post-discharge rehabilitation expenditure would be required to estimate the full economic impact of alcohol-related orthopaedic trauma.

### 4.6. Limitations

The strengths of this study include its eight-year observation period, patient-level exclusion of repeat admissions, prespecified classification of alcohol-related presentations, and confirmation of the principal age-related findings using continuous-age sensitivity analyses.

This study has several limitations. First, its retrospective, single-centre design limits causal inference and may reduce the generalisability of the findings to institutions with different trauma populations, referral pathways, coding practices, and alcohol-consumption patterns. Reliance on routinely collected diagnostic codes and clinical documentation introduces the possibility of incomplete recording and misclassification, which are recognised concerns in studies using administrative health data [11]. Because cohort identification depended on routine diagnostic coding, alcohol use that was not recognised, documented, or coded by the treating team could not be captured. The study therefore cannot estimate the prevalence of alcohol use among all orthopaedic trauma patients. Furthermore, no comparison group of orthopaedic trauma patients without documented alcohol involvement was included; consequently, the present study cannot determine whether alcohol exposure increased the risk of specific injuries, operative treatment, ICU care, mortality, or other outcomes relative to the broader orthopaedic trauma population.

Alcohol-related diagnoses, withdrawal, comorbidities, and injury mechanisms may therefore have been under-recorded. The mutually exclusive alcohol classification represented the predominant documented presentation during admission and should not be interpreted as a measure of consumption, dependence severity, or withdrawal severity. Structured screening using AUDIT-C or PAWSS was not routinely available [16,19], blood ethanol testing was documented in 157 patients (53.4%), and information regarding the quantity and duration of alcohol consumption, timing of the last intake, previous withdrawal episodes, or prior withdrawal seizures was unavailable. Recurrent trauma was identified only when patients returned to the same institution, and the opportunity to record a repeat admission varied according to the year of initial presentation. The exact onset of alcohol withdrawal relative to surgery was not consistently recorded; therefore, the association between documented withdrawal and a longer admission-to-surgery interval cannot establish temporal or causal direction.

The high proportion of unspecified injury mechanisms (38.1%) reduces the precision of the observed mechanism distribution and particularly limits interpretation of less frequently documented mechanisms such as interpersonal violence and transport-related trauma. The administrative annual denominator included all hospital episodes managed within the section rather than unique acute orthopaedic trauma patients, and therefore could not be used to estimate the prevalence of documented alcohol use in the overall trauma population.

ICU involvement was derived from electronically recorded ICU episodes and included both brief perioperative surveillance and prolonged ICU stays. Although episode duration and the temporal relationship to surgery could be evaluated, planned versus unplanned ICU admission and the specific indication for ICU care were not consistently available. Similarly, detailed injury-severity measures were limited. Polytrauma was based on routine clinical documentation rather than a systematically available Injury Severity Score or Berlin definition, and a Gustilo–Anderson grade was not consistently available for all open fractures.

Only ten in-hospital deaths occurred, preventing reliable multivariable mortality analysis. The observed association between documented withdrawal and mortality should therefore be regarded as an unadjusted exploratory safety signal and remains susceptible to confounding by injury severity, comorbidity, infection, frailty, and other clinical factors. The multivariable analyses were also exploratory and were based on routinely collected variables. The proximal femoral fracture model included injury mechanism and fracture pattern arising from the same traumatic event and demonstrated limited calibration; it should therefore be interpreted descriptively rather than causally. Residual confounding may remain because frailty, bone mineral density, nutritional status, medication exposure, functional status, and detailed injury-severity scores were not available. A validated comorbidity index such as the Charlson Comorbidity Index could not be reliably reconstructed from the available routinely coded data. In addition, the relatively low recorded prevalence of osteoporosis should be interpreted as likely reflecting underdiagnosis or undercoding rather than the true prevalence of skeletal fragility in this population.

Finally, the analysis was restricted to the index hospitalisation. Post-discharge mortality, fracture union, infection, reoperation, functional recovery, rehabilitation requirements, readmission, and long-term alcohol-related outcomes could not be assessed. Hospitalisation costs were recorded as nominal values and were not adjusted for inflation, reimbursement changes, implant and consumable prices, or modifications in hospital accounting practices; accordingly, the cost findings should be considered descriptive rather than a formal health-economic evaluation. Psychological outcomes, including symptoms of anxiety, depression, and post-traumatic stress disorder, and access to psychosocial support were not systematically recorded [5]. The clinical case was selected to illustrate a relevant management challenge and should not be interpreted as representing the frequency or typical course of withdrawal-related complications within the entire cohort.

## 5. Conclusions

Documented alcohol use identified a clinically heterogeneous orthopaedic trauma population with distinct age-related injury patterns. Geriatric patients were more frequently affected by low-energy falls and proximal femoral fractures and had a greater burden of recorded comorbidity. Documented alcohol withdrawal was associated with longer hospitalisation, a longer admission-to-surgery interval, and higher unadjusted in-hospital mortality, although causal relationships cannot be established from the present data. In orthopaedic patients, withdrawal-related agitation and unsafe mobilisation may also create specific mechanical safety concerns. These findings support careful assessment of alcohol-related risk at admission, early recognition of withdrawal, and heightened inpatient safety precautions. Prospective multicentre studies using structured alcohol screening, detailed injury-severity measures, and post-discharge follow-up are required to determine whether targeted screening and prevention protocols improve clinical outcomes.

## Figures and Tables

**Table 1:** Demographic, Comorbidity, and Predominant Alcohol-Related Characteristics According to Age Group.

Characteristic	Overall, N = 294	Age 18–64 Years, n = 207	Age ≥ 65 Years, n = 87	Global *p* Value	Holm-Adjusted Post Hoc *p* Value
Age, years, mean ± SD	56.3 ± 13.4	49.7 ± 9.8	71.8 ± 5.9	-	
Male sex	221 (75.2%)	163 (78.7%)	58 (66.7%)	0.038	
Rural residence	194 (66.0%)	140 (67.6%)	54 (62.1%)	0.419	
Recorded comorbidities					
Cardiovascular comorbidity	77 (26.2%)	36 (17.4%)	41 (47.1%)	<0.001	
Chronic liver disease	33 (11.2%)	20 (9.7%)	13 (14.9%)	0.225	
Neurological or cognitive comorbidity	34 (11.6%)	21 (10.1%)	13 (14.9%)	0.238	
At least one selected comorbidity	117 (39.8%)	66 (31.9%)	51 (58.6%)	<0.001	
Predominant recorded alcohol-related presentation				0.014	
Alcohol withdrawal	46 (15.6%)	35 (16.9%)	11 (12.6%)		0.772
Acute alcohol intoxication	80 (27.2%)	66 (31.9%)	14 (16.1%)		0.025
Harmful alcohol use or dependence	150 (51.0%)	95 (45.9%)	55 (63.2%)		0.025
Documented alcohol use without a specific clinical phenotype	18 (6.1%)	11 (5.3%)	7 (8.0%)		0.772

Data are presented as n (%) unless otherwise indicated. SD, standard deviation. Cardiovascular, chronic liver, and neurological or cognitive comorbidities were identified from routinely recorded diagnostic fields. “At least one selected comorbidity” indicates the presence of at least one of these three comorbidity categories and is not a validated comorbidity index. Alcohol-related categories were mutually exclusive and represented the predominant recorded presentation during the index admission. The overall distribution of alcohol-related presentations was compared using Pearson’s chi-square test. Category-specific post hoc comparisons were performed using two-sided Fisher’s exact tests, with *p* values adjusted using the Holm method. Other categorical comparisons were performed using two-sided Fisher’s exact tests. Age was not statistically compared because it defined the study groups.

**Table 2:** Distribution of Injury Patterns, Treatment, and Hospital Outcomes by Age Group.

Characteristic	Overall, N = 294	Age 18 to 64 Years, n = 207	Age 65 Years or Older, n = 87	*p* Value
Injury mechanism				0.001
Same-level or low-energy fall	93 (31.6%)	53 (25.6%)	40 (46.0%)	0.005 †
Falls from height or other specified falls	34 (11.6%)	27 (13.0%)	7 (8.0%)	0.635 †
Road, transport, or bicycle-related injury	28 (9.5%)	23 (11.1%)	5 (5.7%)	0.580 †
Interpersonal violence or other traumatic mechanism	27 (9.2%)	25 (12.1%)	2 (2.3%)	0.028 †
Unspecified mechanism	112 (38.1%)	79 (38.2%)	33 (37.9%)	1.000 †
Open fracture	54 (18.4%)	40 (19.3%)	14 (16.1%)	0.621
Proximal femoral fracture	80 (27.2%)	45 (21.7%)	35 (40.2%)	0.002
Documented polytrauma	22 (7.5%)	17 (8.2%)	5 (5.7%)	0.628
Operative treatment	249 (84.7%)	182 (87.9%)	67 (77.0%)	0.021
Hospital length of stay, days, median (IQR)	8.0 (5.0 to 11.3)	8.4 (5.0 to 12.1)	6.9 (5.1 to 10.5)	0.211
Any recorded ICU involvement	117 (39.8%)	70 (33.8%)	47 (54.0%)	0.002
Prolonged ICU stay, 24 h or longer	19 (6.5%)	15 (7.2%)	4 (4.6%)	0.604
In-hospital mortality	10 (3.4%)	7 (3.4%)	3 (3.4%)	1.000

Data are presented as n (%) unless otherwise indicated. IQR, interquartile range; ICU, intensive care unit. The overall distribution of injury mechanisms was compared using Pearson’s chi-square test. The corresponding effect size was Cramér’s V = 0.245. Category-specific comparisons marked with † were performed using two-sided Fisher’s exact tests and adjusted for multiple comparisons using the Holm method. Binary clinical outcomes were compared using Fisher’s exact test, and hospital length of stay was compared using the Mann–Whitney U test. Proximal femoral fractures included femoral neck, trochanteric, and subtrochanteric fractures. Any recorded ICU involvement included both brief perioperative surveillance and prolonged ICU stays. A prolonged ICU stay was defined as a recorded ICU episode lasting 24 h or longer. The broader road, transport, or bicycle-related category included five bicycle injuries that did not meet the prespecified definition used for the dedicated road-traffic subgroup.

**Table 3:** Exploratory Multivariable Logistic Regression Analyses for Any Recorded ICU Involvement and Proximal Femoral Fracture.

**A. Outcome: Any Recorded ICU Involvement**			
**Predictor**	**Adjusted OR**	**95% CI**	***p* Value**
Age 65 years or older	1.80	1.03 to 3.13	0.038
Male sex	0.72	0.41 to 1.29	0.273
Open fracture	1.38	0.72 to 2.67	0.332
Polytrauma	1.83	0.72 to 4.62	0.203
Proximal femoral fracture	2.96	1.66 to 5.27	<0.001
Alcohol withdrawal	1.21	0.60 to 2.42	0.593
At least one selected comorbidity	1.43	0.85 to 2.42	0.181
**B. Outcome: Proximal femoral fracture**			
**Predictor**	**Adjusted OR**	**95% CI**	***p* value**
Age 65 years or older	1.84	1.02 to 3.31	0.042
Male sex	1.52	0.79 to 2.93	0.21
Same-level or low-energy fall	2.12	1.21 to 3.71	0.009
Harmful alcohol use or dependence	1.21	0.69 to 2.11	0.501
At least one selected comorbidity	1.96	1.13 to 3.42	0.018

Data are presented as adjusted odds ratios with 95% confidence intervals. The ICU model included 117 events among 294 patients. The proximal femoral fracture model included 80 events among 294 patients. “At least one selected comorbidity” indicated the presence of cardiovascular, chronic liver, or neurological or cognitive comorbidity. Reference categories were age 18 to 64 years, female sex, absence of the listed injury or clinical characteristic, and absence of the selected comorbidities.

**Table 4:** Clinical Characteristics, Injury Features, and Hospital Outcomes in the Road-Traffic Subgroup.

Characteristic	Road-Traffic Subgroup, N = 23
Age, years, mean ± SD	48.0 ± 14.7
Age ≥ 65 years	4 (17.4%)
Male sex	20 (87.0%)
Pedestrian	12 (52.2%)
Driver or passenger	4 (17.4%)
Cyclist or other road user	3 (13.0%)
Unspecified road user	4 (17.4%)
Documented acute alcohol intoxication	14 (60.9%)
Open fracture	7 (30.4%)
Polytrauma	10 (43.5%)
Operative treatment	19 (82.6%)
Hospital length of stay, days, median (IQR)	8.5 (6.2 to 20.0)
Any recorded ICU involvement	9 (39.1%)
In-hospital mortality	2 (8.7%)

Data are presented as n (%) unless otherwise stated. SD, standard deviation; IQR, interquartile range; ICU, intensive care unit. Documented acute alcohol intoxication was present in 60.9% of cases, with a 95% confidence interval of 40.8% to 77.8%.

## Data Availability

The data supporting the findings of this study are available from the corresponding author upon reasonable request, subject to approval by the relevant institution and applicable ethical and patient-confidentiality restrictions. The data are not publicly available because they contain potentially sensitive clinical information.

## Abbreviations

The following abbreviations are used in this manuscript:

AUDIT-C	Alcohol Use Disorders Identification Test—Consumption
CI	confidence interval
ICD-10	International Classification of Diseases, Tenth Revision
ICU	intensive care unit
IQR	interquartile range
OR	odds ratio
PAWSS	Prediction of Alcohol Withdrawal Severity Scale
RECORD	Reporting of studies Conducted using Observational Routinely collected health Data
SD	standard deviation
STROBE	Strengthening the Reporting of Observational Studies in Epidemiology
